# UNDERSTANDING AND EXPERIENCING AGEISM: PERSPECTIVES FROM OLDER ADULTS

**DOI:** 10.1093/geroni/igae098.3915

**Published:** 2024-12-31

**Authors:** Aaron Li, Nancy Morrow-Howell, Natalie Galucia, Khrystal Johnson, Brian Carpenter

**Affiliations:** Washington University in St. Louis, St. Louis, Missouri, United States; Washington University in St. Louis, St. Louis, Missouri, United States; Washington University in St. Louis, St. Louis, Missouri, United States; Washington University in St. Louis, St. Louis, Missouri, United States; Washington University in St. Louis, St. Louis, Missouri, United States

## Abstract

Interventions to reduce ageism usually target younger people and service providers, with little research on interventions directed explicitly at older people to identify and resist interpersonal and internalized ageism. This study increases knowledge to guide the development of such interventions by elucidating how older people think about ageism and clarifying motivations to confront it. We also need to know the language older people use to describe ageism and the experiences they report in employment, health care, and family setting. This knowledge is important for designing acceptable and effective anti-ageism programs. We employed a focus group methodology, gathering data on the lived experience of ageism among a diverse group of 50 people over the age of 60. Questions elicited participants’ thoughts about interpersonal and internalized ageism, examples of how they experienced it, and how they reacted. Findings suggest that older people are not clear about what constitutes interpersonal ageism, and internalized ageism is difficult to identify. Instead, participants resorted to talking about their experiences of growing older and frequently mentioned a lack of respect as the hallmark of ageism. Examples of ageism within their families and among younger acquaintances were more common than examples in other domains of daily life. Older age did not rise to the level of importance of other marginalized identities; instead, racism, ableism and homophobia were more central to their experiences of discrimination. Findings suggest that interventions with older people must include consciousness-raising about ageism and its consequences well as common examples and skills to combat it.

